# Zebrafish Models of Cancer—New Insights on Modeling Human Cancer in a Non-Mammalian Vertebrate

**DOI:** 10.3390/genes10110935

**Published:** 2019-11-15

**Authors:** Martina Hason, Petr Bartůněk

**Affiliations:** Institute of Molecular Genetics of the Czech Academy of Sciences, v. v. i. Vídeňská 1083, 142 20 Prague 4, Czech Republic; martina.hason@img.cas.cz

**Keywords:** zebrafish, epigenetics, xenotransplantation, drug screen, pre-clinical cancer model

## Abstract

Zebrafish (*Danio rerio*) is a valuable non-mammalian vertebrate model widely used to study development and disease, including more recently cancer. The evolutionary conservation of cancer-related programs between human and zebrafish is striking and allows extrapolation of research outcomes obtained in fish back to humans. Zebrafish has gained attention as a robust model for cancer research mainly because of its high fecundity, cost-effective maintenance, dynamic visualization of tumor growth in vivo, and the possibility of chemical screening in large numbers of animals at reasonable costs. Novel approaches in modeling tumor growth, such as using transgene electroporation in adult zebrafish, could improve our knowledge about the spatial and temporal control of cancer formation and progression in vivo. Looking at genetic as well as epigenetic alterations could be important to explain the pathogenesis of a disease as complex as cancer. In this review, we highlight classic genetic and transplantation models of cancer in zebrafish as well as provide new insights on advances in cancer modeling. Recent progress in zebrafish xenotransplantation studies and drug screening has shown that zebrafish is a reliable model to study human cancer and could be suitable for evaluating patient-derived xenograft cell invasiveness. Rapid, large-scale evaluation of in vivo drug responses and kinetics in zebrafish could undoubtedly lead to new applications in personalized medicine and combination therapy. For all of the above-mentioned reasons, zebrafish is approaching a future of being a pre-clinical cancer model, alongside the mouse. However, the mouse will continue to be valuable in the last steps of pre-clinical drug screening, mostly because of the highly conserved mammalian genome and biological processes.

## 1. Introduction

In the last four decades, a significant amount of time and money have been invested into the investigation of cancer. Cancer is a collective term for a large number of genetically diverse diseases that share common hallmarks at the cellular and molecular level. The diversity of tumors seems to be one of the biggest challenges for treating cancer patients as the inter-individual differences are enhanced by intratumor heterogeneity. Intratumor heterogeneity is the cellular variability of cancerous tissue and has been found in the vast majority of cancer types. Tumor cells differ in their genomes, transcriptomes, proteomes as well as their epigenomes. Further, cancer cells undergo subclonal evolution during tumor growth. In consequence, cancer cell metabolism, as well as its proliferative and metastatic potential, rapidly changes in time [1,2,3]. Until now, there is no easy way to address the great diversity of cancer malignancies, nor in approaching cancer therapy. Usually, the identification and the targeting of frequent driver mutations is a rational approach to cancer treatment. The field of current cancer research has been innovative in the last years by focusing on tumor cell growth, evolution, and heterogeneity, especially by looking into these processes in live animals. A trend towards targeted therapy and combination therapy has been facilitated by testing in various animal models in vivo [4]. The murine model has been routinely used in cancer research mostly because of the physiological as well as the genetic similarities to human. However, the main disadvantage of mice for cancer research is that it is basically impossible to study early tumor dissemination and changes in the tumor microenvironment at the cellular level. Further, the mouse is not suitable for large-scale small molecule screening. Many of the hurdles posed by unknown tumor driver mutations or treatment resistance could be partially overcome by patient-derived cancer cell xenotransplantation (PDX) followed by whole-animal high-throughput small molecule screening. Another drawback of the murine model, specifically for PDX, is the fact that the tumor graft needs to be transplanted into an immunocompromised adult recipient [5,6,7]. Human cancer research is not limited only to mammalian models [4,8,9].

Fish, as non-mammalian vertebrate models of cancer, are not new to the field. Their advantages for biomedical research are the relatively low-cost maintenance at high numbers of animals, the external development which allows in vivo imaging, and the large number of progeny. The first model of melanoma in fish was established in platyfish (*Xiphophorus*). It was shown that the genetic hybrids of the pigmented platyfish (*Xiphophorus maculatus*) and the non-pigmented swordtails (*Xiphophorus helleri*) spontaneously develop melanoma. This model is one of the earliest animal cancer models [10,11]. Another example is medaka (*Oryzias latipes*), a small freshwater fish which has helped to uncover new aspects of cancerogenesis, again mostly in melanoma pathogenesis [10,12]. Zebrafish (*Danio rerio*) has gained the most attention as a robust animal for studying development and disease. Due to its cost-effective maintenance, high fecundity, fast external development, optical clarity, and small size of the embryos as well as adults, this little fish has become a popular model organism for developmental biology [4,8,9,13,14]. Thanks to the transparency of zebrafish embryos and larvae it is possible to visualize tumor cell growth and dynamics at early stages of cancer development in vivo [15]. A zebrafish genetic strain that maintains much of its transparency throughout adulthood, known as *casper*, has been created as well [16]. Further, the efficiency and relative ease of genetic manipulation for mutant and transgene production makes zebrafish a versatile animal for disease modeling. Major players of human cancer-related pathways have their homologs in the zebrafish genome [17,18,19]. There are well-established zebrafish transgenic lines with fluorescently labeled tissues available that can add new insights into cancer cell growth, dissemination, and tumor microenvironment in real-time [20,21,22]. Aspects of human disease can be recapitulated and followed in vivo in zebrafish at the molecular level because of its highly evolutionarily conserved genes and signaling pathways [23]. In the last decade, human cancer cell xenotransplantation into zebrafish has been developed as well. Thus, zebrafish has joined the mouse as a new model for xenograft assays. The possibility to maintain high numbers of larvae at one place and time makes zebrafish a convenient model for small molecule screening in drug discovery [24,25,26]. This is of high importance in the emerging field of PDX small molecule screening. Zebrafish accelerates the pre-clinical development process as its embryos are suitable for large-scale whole animal screening [19,27,28]. 

Zebrafish is a poikilothermic fish with a preferred temperature around 28 °C. This might be adverse in studies where the mammalian homeostatic temperature would be important. However, in short time periods, zebrafish can tolerate temperatures ranging from 6 to 38 °C [29]. Another drawback of zebrafish is the teleost genome duplication, where there are genes in more than one copy (paralogs). This means that some genes could be redundant in function or that their function could be sub-divided from the ancestral genes’ function. This fact might complicate molecular genetic studies in zebrafish [30,31]. Additionally, there is a lack of commercially available antibodies against zebrafish proteins. This disadvantage is at least partially compensated by the availability of reporter transgenic zebrafish lines [15]. 

In this review we will provide an insight into zebrafish models of cancer, focusing on genetic modeling of cancer in zebrafish, on recent research progress in transplantation studies, and small molecule drug screening models; and on novel approaches in modeling tumor growth in zebrafish, for example by using transgene electroporation in adult zebrafish (TEAZ) [32]. We will further discuss the use of zebrafish in following cancer metastasis real-time in vivo. Metastasis is a process critical in cancer malignancy, therefore we will further look at the role of tumor microenvironment on influencing cancer cells spreading out of the site of primary tumors [33] which was shown in zebrafish xenograft studies [33]. Another issue, which we want to address in this review, is the importance of epigenetic machinery in such a complex matter as tumor biology. Human whole-genome sequencing has revealed recurrent somatic mutations in many genes encoding epigenetic regulators, several of them were found to be associated with specific cancer types [34,35,36].

## 2. Genetic Models of Cancer in Zebrafish

Disease-modeling in zebrafish is versatile and can be approached from many angles, either by creating gene-targeted mutations and stable transgenes or by creating a fish with transient overexpression or downregulation of certain genes. First, forward genetic screens done in zebrafish revealed that the use of common mutagens, such as ethylnitrosourea (ENU) or *N*-methyl-nitrosoguanidine (MNNG), cause the development of various neoplasms, for example, adenoma or rhabdomyosarcoma (RMS) [37,38]. One of the first models of cancer in zebrafish which was found in an ENU screen was the fish with a mutation in the *tumor suppressor 53* (*tp53^M214K^*). *TP53* is the most frequently mutated tumor suppressor gene found in human cancers. These mutant *tp53^-/-^* animals develop malignant peripheral nerve sheath tumor (PNST) which are often recognized as a subtype of sarcoma. PNST was rarely seen in wild-type (WT) fish. The zebrafish phenotype partially recapitulates the situation observed in *TP53* inactivated human patients. They, however, develop a wide array of cancer types in addition to sarcomas, such as breast cancer, brain tumor, or leukemia [39]. A newer zebrafish model with *tp53^del/del^* loss-of-function deletion allele created in the CG1 syngeneic zebrafish strain was described more recently. These zebrafish develop various types of tumors besides PNST, such as leukemia or germ cell tumors, which is more akin to the situation in human patients [40]. 

In consecutive years, many new techniques have emerged for gene manipulation and transgene introduction into the zebrafish genome. All these reverse genetic approaches aim to generate a loss-of-function phenotype or they aim to transfer genes found mutated in human cancer patients into the fish. This could also mean creating a zebrafish model with a mutation in an orthologous gene to a human cancer-related phenotype [41]. It has been shown that zebrafish can develop lymphoma, resembling acute T-cell lymphoblastic leukemia (T-ALL), with lymphoid tissue-specific overexpression (under *rag2* promoter) of the mouse *mMyc* oncogene. This was another implication for the field that zebrafish can indeed acquire tumors similar to mammals [42,43]. Tumor induction was observed also in a *rag2:KRAS^G12D^* overexpressing zebrafish which developed RMS in time [44]. The tumorigenesis followed by Langenau et al. was even more pronounced when initiated in *tp53^-/-^* deficient zebrafish. The developing tumors were transplantable into other zebrafish recipients [42,43]. These studies were the first ones to describe that tumor suppressor genes and oncogenes can recapitulate cancer phenotypes as we know them from patients, in zebrafish. Together with the evidence for evolutionarily conserved drivers of tumorigenesis, this led to the establishment of zebrafish as a model for human cancer pathogenesis. A contemporary model of melanoma in zebrafish has demonstrated the cooperative function of *tp53^-/-^* mutation with the activating mutation in the serine/threonine kinase BRAF [45,46]. This transgenic zebrafish expresses the mutated form of *BRAF^V600E^* most commonly found in human melanoma under the control of the melanocyte-specific *mitfa* promoter. *BRAF^V600E^* on its own is not sufficient to evoke melanoma in zebrafish. Transgenic animals without *tp53^-/-^* mutation form nevi. Nevi are sites with high melanocyte proliferation which do not advance into malignant melanoma [45]. Many transplantation studies have used cancer cells derived from *BRAF^V600E^-tp53^-/-^* zebrafish and we will review them further in Section 3 of this paper. 

*TP53* is often concurrently mutated in human cancers bearing *BRCA2* mutations. The tumor suppressor gene *BRCA2* affects both the meiotic and mitotic cell cycle. Recently, *brca2* mutant zebrafish in a *tp53^-/-^* background were examined for cell cycle arrest and genomic stability. This model, as it is not embryonically lethal compared to many BRCA2 mouse models, allows for in vivo studies in adult animals [47]. In *brca2* mutant zebrafish, it was previously shown that there is an increased incidence of benign testicular tumors [48]. Concurrent mutations of *brca2/tp53* led to soft tissue sarcomas, predominantly to PNSTs. Surprisingly, *brca2* mutation in females significantly reduced the survival rate after they have developed tumors compared to males with the same genotype. This study further supports the link between *brca2* mutation and cancer aneuploidy with poor survival prognosis [47].

Melanoma has been extensively studied in zebrafish since the first description of the *BRAF^V600E^* model. Melanoma emerges in a form of transformed melanocytes, which are cells derived from the embryonic neural crest and produce pigment. This disease is commonly driven by mutations in *BRAF* and *RAS* in human patients [49]. Melanomic lesion initiation and the mechanism of sporadic melanoma formation was evaluated in zebrafish *crestin:EGFP* expressing embryos and in adults. In embryos, *crestin* is expressed in neural crest cell progenitors and it is re-expressed in melanoma tumors of adult fish. Neural crest cells were shown to be a key element in melanoma initiation in the *BRAF^V600E^*-*tp53^-/-^* zebrafish. [50]. RAS signaling is extensively studied in zebrafish as well. There is a zebrafish model of Costello syndrome driven by *HRAS* mutation derived from human patients (*HRAS^G12V^)*. These fish develop craniofacial and spinal abnormalities. Older fish harboring this mutation were prone to tumor formation, including lymphoma, melanoma, or sarcoma [51]. This model has been further upgraded by the Gal4–UAS system and by the melanocyte-specific expression of *HRAS^G12V^* under the *kita* promoter. The transgenic fish start to develop tumor masses by 2–4 weeks of life so the progress of the disease is relatively fast. Adult tumors show similarities to human melanoma and they are transplantable. This is in contrast to *mitfa* expressing melanocyte progenitors which form melanoma less efficiently in the same Gal4–UAS setup [52]. Another type of *BRAF^V600E^* driven cancer was characterized more recently in zebrafish. This model of thyroid carcinoma was described in transgenic fish expressing *BRAF^V600E^* in thyrocytes, under the expression of *thyroglobulin promoter* (*tg*) [53]. Treatment with MEK and BRAF inhibitors suppressed the oncogenesis and restored normal thyroid morphology. The authors propose in this study a novel potential target responsible for *BRAF^V600E^* driven thyroid follicle transformation in a *TWIST2* zebrafish orthologue—*twist3*. *twist3* is an important transcriptional regulator of epithelial-to-mesenchymal transition (EMT)—a critical process in tumorigenesis, in the acquisition of tumor resistance, and in metastatic spread of tumor cells out of the primary tumor site [33]. Inactivation of this gene led to the suppression of *BRAF^V600E^*-induced effects and led to thyroid morphology restoration and rescued hormone production [53]. Previously, MITF, a melanocyte-specific transcription factor, has been found to be important in melanoma pathogenesis. The inhibition of its activity leads to a dramatic regression in melanoma growth [54]. Recently, the effect of constitutively activated *HRAS^G12V^* on microRNAs (miRNAs) expression level was studied. The transgenic *HRAS^G12V^* zebrafish develops different types of cancer, however, the authors focused on melanoma onset and progression. Activated RAS signaling was found to promote the expression of six different miRNAs. Among them, the most interesting miRNAs are targeting the *jmjd6* gene. *jmjd6* was found to be a critical player in zebrafish melanoma pathogenesis as its increased expression was correlated to more aggressive phenotypes [55]. Zebrafish has also been used to evaluate the effects of mutated *RAS* in the induction of RMS. A mosaic transgenic zebrafish over-expressing the human mutated version of *KRAS^G12D^* under the *rag2* promoter developed RMS in nearly 50% of cases until adulthood [44]. However, mutation of *KRAS* in human patients leads most often to pancreatic adenocarcinomas. *ptf1a:KRAS^G12V^* expressing zebrafish was shown to develop invasive exocrine pancreatic cancer which partially resembled the carcinoma found in human [56]. Recently, there has been an update to this study which presents a zebrafish model with inducible expression of *KRAS^G12V^* that highly recapitulates human pancreatic neoplasia leading to pancreatic adenocarcinoma [57]. 

Zebrafish has proven to be a good model for the study of hepatocellular carcinoma (HCC). HCC is the prevailing type of liver cancer worldwide. It has been shown in zebrafish that the liver-specific expression of the human ribose-5-phosphate isomerase A (*RPIA*) under the expression of *fabp10a* promoter can mediate hepatocarcinogenesis. These transgenic zebrafish develop HCC. Further, β-catenin signaling is activated which in the end elevated the expression of downstream target genes. Levels of phosphorylated ERK were also elevated in the livers of RPIA transgenic fish. Combination therapy with β-catenin and ERK inhibitors synergistically reduced RPIA-induced cellular proliferation in zebrafish. [58] *RPIA* was found to be a valuable therapeutic target [58]. Many recent studies are looking at the pathogenesis of *KRAS*-driven cancer. A zebrafish model with inducible expression of a mutated version of *KRAS^V12^* in the intestine (under the *ifabp* promoter) developed tubular adenoma of the intestine until adulthood [59]. The effects of cancer cachexia, a syndrome affecting cancer patients, which can result in weight loss, muscle wasting, and is predictive of low survival, was studied in an inducible *KRAS^G12V^*-driven HCC zebrafish model [60]. The effects of overfeeding in these zebrafish were striking. Overfeeding accelerated cancer progress and in the end leptin, an obesity hormone, was found to be upregulated in the hepatocytes of overfed groups with carcinoma. Knockout fish lacking the leptin receptor had better survival rates in HCC as their WT siblings. Chemical targeting of the leptin receptor also increased the survival rate of tumor-bearing fish [60]. Another interesting study has shown that sex hormones have an effect on HCC pathogenesis with males being more susceptible to HCC development as well as having a higher mortality rate than females [61]. A transgenic zebrafish expressing the double oncogene *Myc/xmrk* developed a severe HCC with different progression in males and females. The effects of androgen and estrogen treatment were tested. Androgen could promote cell proliferation and estrogen had an inhibitory effect on cancer cell growth and therefore might have a protective role in HCC [61]. The role of *cyp7a1* in tumor-liver cross-talk was studied in a *kras^G12D^*-induced zebrafish model of intestinal cancer. *kras^G12D^* expression in the posterior intestine resulted in the formation of intestinal tumors which led to liver inflammation, hepatomegaly, and death. This was the result of defective metabolism as anomalies in *cyp7a1* expression can lead to altered cholesterol–bile alcohol flux in zebrafish. This is an illustration of the importance of tumor–organ interactions and generally of the importance of metabolic homeostasis in tumorigenesis [62]. From all of the above-mentioned studies, it is evident that RAS signaling defects are common in various types of cancer and the oncogenic activity of RAS is not limited only to melanoma or sarcoma. BRAF and RAS mutations seem to be mutually exclusive in cancer, however, there are rare cases when these players of the same signaling pathway coincide, which might be interesting for further studies [63,64]. The above- mentioned studies have shown that zebrafish is indeed a reliable model to describe cellular as well as molecular mechanisms of malignancies caused by recognized tumor suppressors and proto-oncogenes such as TP53, BRAF, or RAS. 

Hematopoietic programs are strikingly well conserved between human and zebrafish, making it possible to study hematopoietic diseases in fish. Many models of leukemia have already been established in zebrafish. Most of them are based on known human mutations, deletions, or translocations [17,65]. The individual types of leukemia are so diverse, that many of the factors affecting the onset and progression of these types of cancer are still unknown. There have been many studies done in zebrafish since the first model of *Myc*-induced T-ALL [42]. A Cre-lox regulated conditional model of zebrafish T-ALL was developed because the original *rag2-mMyc* expressing zebrafish developed severe disease phenotypes and typically died around three months of age [66]. The underlying causes of myeloid and lymphoid malignancies are very diverse and our understanding of their disease mechanisms is incomplete. Therefore, it is of high interest to have reliable animal models which would allow to better understand the molecular pathogenesis of hematopoietic malignancies. There are classic models of myeloid leukemia in zebrafish, based on chromosomal rearrangements known from humans as well as murine models, based on oncogene mutations or oncogene overexpression [67]. The fusion of *AML1* with *ETO* is one of the most commonly found fusions in acute myeloid leukemia (AML). A zebrafish model with inducible embryonic overexpression of *AML1-ETO* recapitulates the phenotype observed in human patients [68]. Myelo-erythroid progenitor cells are re-programmed and the accumulation of granulocytic cells is observed in this model of AML. The phenotype was treatable and reverted to normal state after the use of Trichostatin A, a histone deacetylase inhibitor [68]. Looking at the classic fusion protein of *AML1-ETO* in another zebrafish study revealed the functional effect of *TLE1* and *TLE4* loss in AML. The authors have found out that *TLE* proteins might have a tumor-suppressive role in the development of myeloid leukemia [69]. In another transgenic zebrafish model of AML, the fusion protein of *MYST3-NCOA2* is expressed under the myeloid cell-specific promoter *spi1*. Only a small amount of fish, about 1%, developed myeloid leukemia until adulthood. Immature myeloid blast cells were accumulating in the kidney of diseased animals [70]. All of the above-mentioned studies were among the first ones to describe AML in the zebrafish model. 

Various fusions of *TEL-JAK2* were found in human patients of myeloid or lymphoblastic leukemia. The expression of *tel-jak2a* fusion protein can disrupt normal embryonic hematopoiesis in zebrafish [71]. Different forms of the zebrafish *tel-jak2a* fusion protein were overexpressed in zebrafish myeloid cells. The authors were able to distinguish two phenotypes when they used two distinct versions of *tel-jak2a* fusions in zebrafish. One phenotype was similar to T-ALL and the other one to atypical chronic myelogenous leukemia (CML). The effects of two different types of *TEL-JAK2* fusions generally found in human patients were compared in the zebrafish *tel-jak2a* model. This study has shown that different types of fusions of the same genes can lead to corresponding phenotypes in zebrafish (ALL and CML respectively), as observed in human patients [72]. 

**Figure 1:**
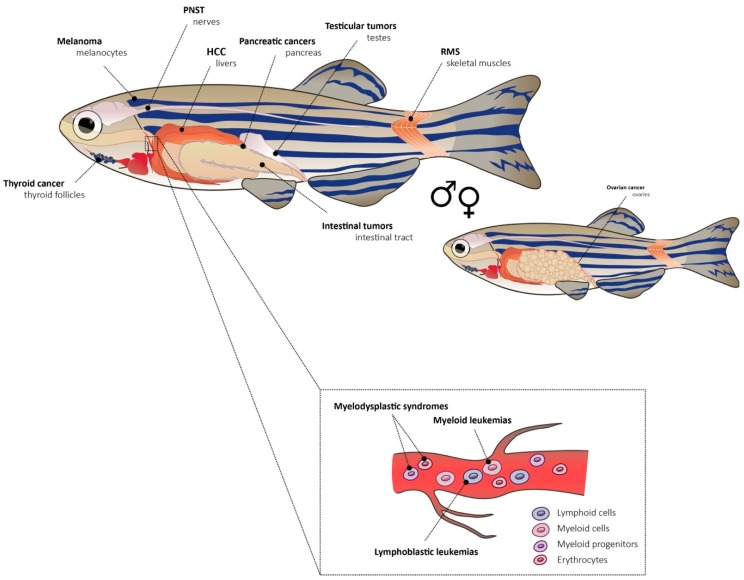
Zebrafish models of cancer. Zebrafish develops cancer phenotypes similar to human cancer in different tissues and organs. All of these cancer types and their zebrafish models are discussed in Section 2. Genetic models of cancer. PNST—peripheral nerve sheath tumor; HCC—hepatocellular carcinoma; RMS—rhabdomyosarcoma; ♂—male; ♀—female.

Receptor tyrosine kinases are important players in hematopoiesis. FMS-like tyrosine kinase 3 (*FLT3*) is crucial in hematopoietic stem and progenitor cells and it plays a role in the development and differentiation of hematopoietic stem cells, dendritic cell progenitors, B-cell progenitors, and natural killer (NK) cells. An internal tandem duplication (ITD) of *FLT3* is found in about 30% of AML patients [73]. The overexpression of human *FLT3*-ITD and *FLT3*-TKD in zebrafish leads to a leukemic phenotype with expanded myelopoiesis during early embryogenesis. The main cell type expanded was monocyte-like, which is typical for AML [74]. A model of myelodysplastic syndrome (MDS) where zebrafish *tet2* (ten-eleven translocation methylcytosine dioxygenase 2) was disrupted, mimics a frequently observed loss-of-function mutation found in humans. These zebrafish developed normally but progressive dysplasia of myeloid progenitors appeared with age [75]. *TET2* encodes a member of the *TET* family of DNA methylcytosine oxidases which mediate demethylation of DNA within genomic CpG islands. The disease developed from pre-myelodysplasia to MDS in adult kidney marrows. The fish had first decreased numbers of erythrocytes and expanded numbers of myelomonocytes and progenitor cells in the marrow but normal peripheral blood counts. With age, they progressed to full MDS with decreased counts of erythrocytes in blood [75]. All the discussed genetic models of cancer with their exact genotypes are summarized in Table 1. A representation of cancer types discussed in this section is depicted in Figure 1 with individual sites of tumors within an adult zebrafish. Further models of tumorigenesis [8,19,76] and leukemia in zebrafish are compiled in other review papers [65,67,77,78,79].

With this extensive list of transgenic and mutant zebrafish models of cancer, we are not aiming to be fully comprehensive. It is merely a broad demonstration of the fact that nearly any cancer type, from carcinomas through melanoma to leukemia, could be studied in zebrafish. All in all, with properly combining tumor suppressors/oncogenes or their mutated version with tissue-specific promoter expression it is possible to generate cancer in zebrafish which is often closely resembling human cancer phenotypes at the histological and molecular level. 

### 2.1. Zebrafish and New Methods for Cancer Modelling

In this section, we will discuss in more detail the most popular and widely used gene manipulation techniques which were engaged in the majority of above-discussed zebrafish cancer-modeling studies [80,81,82,83,84,85,86]. In zebrafish it is possible to perform forward and reverse genetic screens and directly assess the role of various genes in cancer related phenotypes [87]. Currently, the most widely used techniques for zebrafish gene manipulation are antisense morpholino oligonucleotides (MOs) [83], ZFNs (zinc finger nucleases) [84], TALENs (transcription activator-like effector nucleases), [85] and the CRISPR (clustered regularly interspaced short palindromic repeats) system [86].

MOs are small synthetic oligonucleotides which are able to block mRNA translation in vivo. Zebrafish MO gene knockdown phenotypes were extensively compared to knockout phenotypes over the years. There is a discussion about off-target effects of MOs [88,89] and the fact that they often do not fully copy phenotypes of genome-edited mutants generated by TALENs [90] or CRISPRs [88,91] is concerning. Despite these challenges, MOs are still used in the zebrafish community and with proper validation and with the utilization of appropriate controls they can facilitate the generation of large numbers of experimental embryos really fast. The accepted rule today is to confirm the MO specificity by either repeating the knockdown in *tp53^-/-^* mutant embryos or to simultaneously knockdown *tp53* in morphants [92]. Another substantial drawback of MOs, however, is that they are active only in a short time frame of early embryonic development until they get diluted out [83].

The use of site-directed nucleases is convenient for multiplex gene targeting. This way, the often- complicated disease genotypes could be created in one round of genome editing. Further, it is possible to create not only knock-outs and loss-of-function alleles but also knock-ins, where whole open reading frames can be inserted into the zebrafish genome [93]. As the first tool for targeted mutagenesis in zebrafish ZFN was successfully used in the example of the *golden* gene disruption. The resulting homozygous mutant embryos lacked pigmentation. This study showed that ZFN may be applicable to general gene disruption in zebrafish [84]. There were consequent studies which successfully employed ZFN in targeted mutagenesis. A zebrafish model of neurofibromatosis 1 (NF1) was generated by ZFN targeting of *nf1a* and *nf1b* genes. The mutant embryos exhibited similar phenotypes to the ones observed in NF1 patients, as oligodendrocyte hyperplasia and melanophore hypoplasia [94]. Another example of ZFN utilization in zebrafish is the model of MDS generated by mutating *Tet2* discussed above in Section 2 [75].

TALEN has been overly popular for almost a decade. It was actually in zebrafish where it was shown for the first time that TALENs are able to produce heritable gene disruptions in the vertebrate genome [85]. Bedell et al. [95] updated the TALEN system and created and tested its capabilities to effectively edit the zebrafish genome. *cadherin 5* (*cdh5*) mutant zebrafish was created. It had vascular defects, cardiac edema, and loss of circulating blood cells [95]. An important fact is that TALENs can create mutations in somatic tissues at a high success rate, including bi-allelic mutations. This fact was utilized in a proof of principle study which aimed to analyze the role of somatic mutations of the retinoblastoma (*rb1)* tumor suppressor gene. Genetically mosaic adult mutants developed tumors mostly in the brain. Homozygous germline mutants of *rb1* are embryonically lethal, therefore it is desirable to study the aspects of its somatic inactivation [96]. Brain tumor models, including PNST and medulloblastoma, a type of frequently occurring pediatric brain cancer, were created with the TALEN technology. The *cdkn2a/b* gene was inactivated in the zebrafish *tp53^-/-^* background which led to an acceleration in PNST development. The authors also further examined the role of *rb1* somatic inactivation in *tp53^-/-^* background. Interestingly, these mutants developed medulloblastoma-like brain tumors specifically [97]. A complete loss-of-function *tp53^del/del^* zebrafish deletion mutant was created by TALEN. These animals develop various types of tumors, including PNST, angiosarcoma, leukemia, or germ cell tumors. This is in contrast to the established *tp53^-/-^* mutant with the *tp53^M214K^* point mutation and it could be explained by the different nature of these two mutations. Tumor onset and pathogenesis might differ based on the nature of *tp53* mutation [40].

The CRISPR/Cas9 technique has evolved quite rapidly in the last five years and has been widely used in the field of zebrafish disease modeling. The Cas9 endonuclease recognizes specific DNA sequences in an RNA-dependent manner. The guide RNA (gRNA) is engineered in a way that it interacts both with the Cas9 enzyme as well as it binds and targets specific parts of genomic DNA [86,98]. A comprehensive study called CRISPRscan provided insight into the mutagenic activity of the CRISPR/Cas9 system in vivo in zebrafish. The study looks at the stability of sgRNAs (single gRNAs), at the specificity of recognized genomic target sequence, and finally at the use of truncated sgRNAs as an efficient alternative to regular sgRNAs [99]. Zebrafish disease models created by CRISPR-based approaches are numerous and widely used today. It is possible to target multiple genes simultaneously with high efficiency. However, the possibility of off-target activity has to be always considered [100]. A recent study of Ablain et al. [101] focused on the identification of cancer driver genes in melanoma. There are still some incompletely described genetic subtypes of melanoma. Specifically, for example, the “triple wild-type” melanoma which lacks mutations in either of the genes usually found mutated - *BRAF*, *NRAS*, and *NF1* genes. Mucosal melanoma, which is discussed in this study, is characterized by genomic instability and a heterogeneous set of mutated genes found in patient samples. *SPRED1* (sprouty-related, EVH1 domain containing protein 1) loss was found as a new driver in mucosal melanoma and the majority of cases with *SPRED1* loss were genetically “triple wild-type” tumors. To evaluate the function of SPRED1 in vivo, zebrafish was used and a new CRISPR-based platform, termed MAZERATI (Modeling Approach in Zebrafish for Rapid Tumor Initiation), was utilized. This system uses two MiniCoopR vectors, one with Cas9 and gRNA expression and the other one expressing the oncogene of interest [101]. One of the main drawbacks of CRISPR/Cas9, and also of the other site-directed nucleases mediated mutagenesis, is the time needed for breeding germline mutants to get a stable line. To be able to screen for loss-of-function phenotypes in F0 founder animals the mutagenesis efficiency would have to be close to 100%. Scientists have tried to overcome this hurdle by assembling Cas9 protein with sgRNA into a ribonucleoprotein complex (RNP) in vitro before injecting it into the cell of zebrafish embryos. This approach led to high mutagenesis rates in target genes with no significant off-target mutagenesis detected. The so-called crispants maintain highly specific mutant phenotypes, however, unpredictable mosaic allele combinations could occur which can hinder phenotype readouts [102]. There is another study which described a similar approach of creating F0 knockout mutants with Cas9 RNP complexes. The authors injected redundant sets of RNPs targeting a single gene and have screened 50 transcription factor genes with this system. In around 90% of F0 embryos, knockout phenotypes were observed. Around 17% of the embryos had morphologic defects indicating toxicity and possible off-target effects but these levels of toxicity were claimed to be acceptable [103].

Novel types of Cas enzymes have been recently discovered in bacterial strains, such as the Cas12a (Cpf1) enzyme. The benefit of Cpf1 is its greater specificity, that it can process a guide RNA array (crRNA) and that a single targeting guide RNA is shorter, compared to Cas9 sgRNAs [104,105]. Cpf1 from *Lachnospiraceae bacterium* (LbCpf1) has been successfully used in zebrafish for genome editing. This enzyme is fully active at 28 °C [106]. New versions of Cas have been developed in vitro, for example the dead version of Cas (dCas). dCas is not enzymatically active but it can be coupled with transcriptional activators (VP64, a synthetic tetramer of the Herpes Simplex Viral Protein) or repressors (KRAB, Kruppel-associated box protein domain). This way it is possible to use CRISPR/Cas for gene up-regulation or down-regulation [100,107].

The conventional zebrafish cancer models, created by either of the above-mentioned techniques, are done by injecting nucleic acids into one-cell stage embryos. There are certain difficulties with addressing cancer development and pathogenesis in these transgenic and mutant animals. In some cases, the onset and localization of developing tumors are not biologically accurate. Furthermore, tumor spread and evaluation of metastases could be difficult. Some of these drawbacks of the genetically engineered models could be addressed with the technique of cancer cell transplantation into zebrafish embryos and adults [19]. We will discuss these approaches in Section 3 of this article. There is, however, a new exciting method, referred to as transgene electroporation in adult zebrafish (TEAZ), which has been recently developed and used for site-specific de novo tumor initiation in zebrafish adults [32]. With this technique it is possible to inject DNA constructs, containing tissue-specific promoters and genes of interest, into adult tissue. The authors have created a model of aggressive melanoma where the tumor onset took only about seven weeks, compared to four months in conventional models. The versatility of TEAZ has been tested in other tissues such as the heart or the brain. This technique is invaluable, as it is rapid and the expression of genes of interest can be spatially and temporally controlled in adult zebrafish [32].

### 2.2. Zebrafish Cancer Models and Epigenetics 

Epigenetic regulators are important factors in development and disease as they regulate gene activation and inhibition. Epigenetic information is reversibly written in the chemical modifications of DNA bases as well as in histone proteins in nucleosomes. The epigenetic machinery consists of transcription factors and chromatin modifiers which regulate gene expression. The disruption of epigenetic mechanisms was shown to be among key drivers of various types of cancer. This dysfunction can be caused by mutations of genes encoding epigenetic regulators. Misbalance can also be caused by exposure to external or internal factors, such as nutrition or inflammation, which can further affect the stability of epigenetic marks [108,109]. Human whole-genome sequencing has revealed recurrent somatic mutations in genes encoding epigenetic regulators, many of them were found to be associated with cancer. In many cases, mutations of epigenetic regulators are the so-called driver mutations which are often present in a specific cancer type. These drivers contribute to cancer pathogenesis to a great extent. In other cases, somatic mutations of epigenetic regulators can represent an additional hit in tumorigenesis which is primarily caused by a mutation of a proto-oncogene or a tumor suppressor [34,110].

Many of the classical models of leukemia, comprising fusion genes, involve an epigenetic regulator as one of the fused genes. This causes deregulation of hematopoiesis and the consequent malignant transformation of cells. For myeloid malignancies, the most prevalent mutations in epigenetic regulators are in *TET2*, isocitrate dehydrogenase 1 and 2 (*IDH1*, *IDH2)*, additional sex combs-like 1 (*ASXL1*), enhancer of zeste homolog 2 (*EZH2*), and DNA methyltransferase 3A (*DNMT3A*) [111,112]. As most downstream actions of epigenetic regulators are in theory, reversible, they represent a priority target for therapeutic screens. In the case of solid tumors, there is also evidence about the role of epigenetic regulators in their pathogenesis. There are many cases where the dysfunction of the same epigenetic regulator has a different role in a wide variety of cancers. The development and implementation of a wide array of epigenetic regulator enzyme inhibitors progressed quite fast in the last years. However, it is still a long way to find specificity and selectivity as well as to overcome the pleiotropic effect of these inhibitors outside of the tumor tissue [113,114,115]. 

The function of epigenetic regulators is usually not exclusive for a specific tissue, nor cancer type, as mentioned above. Ectopic overexpression of *EZH2* in a benign prostate cancer cell line was shown to act as an oncogene and is correlated with poor prognosis of the disease [116]. On the other hand, the role of EZH2 in myeloid and lymphoid disorders seems to be tumor suppressive. *EZH2* mutations were associated with poor prognosis. EZH2 interacts with other proteins which together form the polycomb repressive complex 2 (PRC2). This suggests that diverse mutations can have different effects on the function of the whole complex, hence the broad number of phenotypes caused by *EZH2* mutations [117]. Interestingly, it has been shown that gain-of-function mutations in the *TP53* tumor suppressor led to a broad upregulation of chromatin remodeling enzymes, for example members of the COMPASS methyltransferase pathway, resulting in an increase of histone acetylation and methylation [118]. A genetic screen done in zebrafish identified the histone H3 lysine 9 histone methyltransferase, *SUV39H1*, out of other chromatin-modifying factors, as a tumor suppressor. This methyltransferase was shown to be important in suppressing RMS formation in *rag2*-*hKRAS^G12D^*-induced tumors [80]. The tumor-suppressive role of *SUV39H1* has been shown before in a mouse model of retinoblastoma [119] and this tumor-suppressive role is recapitulated in zebrafish, supporting its importance and evolutionary conservation [80]. The importance of the histone methyltransferase *SETDB1* was shown in a zebrafish model of *BRAF^V600E^ tp53^-/-^* melanoma. The zebrafish used in this study had an additional mutation in *mitfa* and therefore was lacking melanocytes as well as melanoma. The growth of melanocytes in *mitfa:BRAF^V600E^; tp53^-/-^; mitfa^-/-^* fish was rescued with the miniCoopR vector system which simultaneously expressed candidate human genes of interest [81]. *SETDB1* significantly enhanced the aggressiveness of melanoma and accelerated tumor onset. HOX genes were shown to be dysregulated in the presence of upregulated *SETDB1* so *SETDB1* acts as an oncogene in melanoma pathogenesis [81]. Novel epigenetic drug targets have been found thanks to a transgenic zebrafish model of AML expressing the human *NUP98-HOXA9* (*NHA9*) fusion oncogene. These embryos are anemic with myeloid cell expansion and adult animals develop myeloproliferative neoplasms. *NHA9* function depends on downstream activation of *meis1* (*myeloid ecotropic integration site 1*), of the COX (cyclooxygenase) pathway, and of *dnmt1* (*DNA (cytosine-5)-methyltransferase 1*) [82]. The authors used a combination of inhibitors targeting DNMT or COX together with HDAC (histone deacetylase). A strategy for an alternative epigenetic-based treatment of aggressive AML is suggested in this study with zebrafish as a prospective pre-clinical disease model [82]. A study about oncogene drivers in the *rb1* zebrafish model of embryonal brain tumors has found new epigenetic drivers of oncogenesis. Specifically, the authors found more than 170 chromatin regulating genes to be differentially expressed in *rb1* tumors, for example, *histone deacetylase 1* (*hdac1*) and *retinoblastoma binding protein 4* (*rbbp4*) [36]. Zebrafish models of epigenetic regulators involved in cancer are summarized in Table 2.

## 3. Transplantation Models—Allografts and Xenografts

Tumor cell transplantation is a relevant method for tumor invasiveness assessment. Tumor cells from a donor can be grown in a recipient of the same species (allograft) or another species (xenograft). Zebrafish develops cancer, which is invasive and transplantable, in a similar way to humans. Thanks to the natural transparency of zebrafish embryos, and the transparent *casper* strain, it is possible to track and image cancer cell growth in vivo [16]. Zebrafish embryos can engraft transplanted cancer cells until the onset of the adaptive immune system at around seven days post fertilization (dpf). Further maturation of cells leading to immune competence can last until two to four weeks post fertilization [24,28,120]. After surpassing this time window, there are a couple of ways how to deal with the high frequency of transplant rejection when introducing foreign cells into a host organism. The first technique, still widely used, is the sub-lethal irradiation of recipient animals to deplete immune cells in zebrafish [121,122] and mouse [123]. The second way how to introduce and transplant cells from a donor to recipient is to use genetically immunocompromised animals as recipients. This approach has been successfully used for a long time in mouse [5] and the first immunodeficient zebrafish was used for the first time by Tang et al. [124]. These models will be further discussed in the following sections.

### 3.1. Zebrafish as a Model for Allogeneic Transplantation

The first study describing *mMyc*-induced T-cell leukemia in zebrafish has also shown the possibility to transplant zebrafish leukemic cells into γ-irradiated adult WT zebrafish [42]. Apart from γ-irradiation, it is also possible to decrease the immune response of zebrafish by dexamethasone treatment [125] and there is also a clonal syngeneic zebrafish strain (CG1) which was published as a model for allogeneic tissue and cell engraftment [126]. Transplantation of T-ALL derived cells into syngeneic zebrafish revealed that up to 16% of the transplanted cells are self-renewing and have tumor-initiating potential [127].

Another approach to graft introduction is to employ genetically immunocompromised animals, which lack some or all of the functional cells of the adaptive immune system. Typically, the murine severe combined immunodeficiency (SCID) model has been used for these purposes [5] and other immune-deficient murine models as well [128]. In zebrafish, there are few published immunodeficient strains. The first established immunocompromised zebrafish model harbors a frameshift mutation at amino acid E450 of the *recombination activating gene 2* (*rag2*) gene, resulting in a premature stop codon (*rag2^E450fs^*). These fish lack mature T-cells and have a reduced number of B cells. The authors used this mutant fish for allograft transplantations into adult fish [124]. Later, a comprehensive study was published about allografts of T-ALL, embryonal RMS, and melanoma in *rag2^E450fs^* zebrafish in the transparent *casper* background. The authors optimized cell transplantation and were able to follow fluorescently labeled cancer cell growth, tumor formation, and metastasis in adult recipients [129]. A further study published new zebrafish immunodeficient models with affected T-cells, B-cells, and presumptive NK cells. Two zebrafish strains were created in this study. The first, containing a frameshift at aspartic acid residue 3612 resulting in a premature stop codon of the *DNA-dependent protein kinase* (*prkdc^D3612fs^*), resulted in a lack of T- and B-cells. The other, containing a frameshift at proline residue 369 which leads to a premature stop codon in *janus kinase 3* (*jak3^P369fs^*), resulted in a lack of T-cells and NK cells [130]. Both mutants were crossed into the *casper* background to allow better options for in vivo imaging of single cells. However, low survival rates were observed after transplantation to the *jak3^P369fs^* mutant zebrafish. On the contrary, *prkdc^D3612fs^* mutants are able to engraft allogeneic transplants with high efficiency and survive at high numbers. Unfortunately, probably due to still functional NK cells in this mutant, xenografts of human melanoma, breast cancer nor pancreatic adenocarcinoma cells were able to survive, and their growth in the adult mutant fish regressed a week post-transplantation [130].

In recent years a zebrafish melanoma cancer cell line ZMEL has been widely used to rapidly study melanoma pathogenesis and inhibition. ZMEL was derived from melanomas of the *mitfa*-*BRAF^V600E^ tp53^-/-^* transgenic fish [131]. ZMELs have been since used for transplantation studies to assess melanoma pathology and metastatic behavior in zebrafish [132]. Hyenne et al. have recently published a paper focusing on the fate of tumor extracellular vesicles (EVs) derived from ZMELs. They show that EVs can be tracked in vivo in zebrafish and that they activate macrophages and promote metastases [133]. Zebrafish models of allogeneic transplantation are summarized in Table 3.

### 3.2. Zebrafish Xenotransplantation Model for the Evaluation of Cancer Progress and Metastasis 

Zebrafish as a tool in human cancer xenotransplantation studies could overcome some of the drawbacks of the murine model. The main benefits of zebrafish are most prominent when using embryonal stages for xenotransplantation. With the small-sized transparent embryos lacking a mature immune system, it is possible to transplant and track high numbers of animals. This fact is a powerful reason for the utilization of zebrafish as a pre-clinical screening model which could lead to patient-derived cancer cell xenotransplantation and to new options for personalized medicine [19]. Most of the recent transplantation studies in zebrafish use embryonal stages of 48 hours post fertilization (hpf) as the stage for transplantation. However, some of the first zebrafish xenograft studies were done in the blastula stage of the embryo. Transplanted melanoma cells survived, divided, stayed in de-differentiated stage but did not form tumors in zebrafish embryos. This was the first observation of human melanoma cells in zebrafish [134]. In a study utilizing the same type of melanoma xenotransplantation into zebrafish blastula, the authors compared different types of human cutaneous and uveal melanoma cancer cell lines. They found out that aggressive melanoma cells secrete Nodal. The expression of Nodal correlated with melanoma aggressiveness and progression, and caused developmental defects of the zebrafish embryo [135]. Haldi et al. optimized the parameters for zebrafish xenotransplantation where they propose the 48 hpf developmental stage as the best for transplantation. At this stage, developmental cell migration is finished, therefore cancer cell migration after injection is likely to be an active process. Human melanoma cells together with other types of cancer cell lines, which they transplanted into zebrafish, were able to survive and formed tumors in the embryo [136]. The site of transplantation might be variable but usually it is the yolk sac, cardinal vein, Duct of Cuvier, or the hindbrain. Depending on the site of transplantation different phenotypes of tumorigenesis could be followed, for example, cancer cell invasion, extravasation, and metastasis [137], or the interaction of cells with the tumor microenvironment [138]. The importance of increased incubation temperature of zebrafish embryos after xenotransplantation should not be discounted as temperature was shown to be critical for achieving efficient cancer cell proliferation rates [139]. The first study which showed that zebrafish could be used for human PDX provided a simple and fast method for testing the metastatic behavior of primary cancer cells. The authors used a whole set of cancer cell lines as well as primary human cancer cells from pancreas, colon, and stomach carcinomas. Tumor cell invasion and micrometastasis were evaluated and followed in vivo also thanks to the *fli1:eGFP* zebrafish strain with fluorescently labeled vasculature [140]. It is obvious from the studies mentioned above, that zebrafish embryos can engraft human cancer cells and recapitulate disease pathogenesis. Therefore, PDX studies in zebrafish are emerging more often and they can be valuable in accelerating the design of personalized cancer therapy.

Zebrafish has been used to study the tumor microenvironment from the point of tumor-induced angiogenesis. Tumor neovascularization is an important element in tumor growth and metastatic spread. Cancer cells are releasing angiogenic growth factors into the tumor environment which promote neovascularization. Zebrafish embryos enable real-time in vivo visualization of the first steps of tumor neovascularization. *VEGFR2:G-RCFP* transgenic zebrafish embryos with green endothelial cells were transplanted with tumorigenic FGF2-overexpressing mouse aortic endothelial cells and various human cancer cells. The authors showed neovascularization at the tumor site and were able to discriminate between highly and poorly angiogenic tumor cells. The site of transplantation was by the yolk sac, close to the subintestinal veins (SIVs) which originate from the duct of Cuvier. These results were comparable to the effects seen in mouse [141,142]. The contribution of *VEGFR2*^+^ individual endothelial cells to the formation of the tumor vascular network was assessed in the *flk1:EGFP* transgenic zebrafish with fluorescently labeled blood vessels. SU5416, a VEGFR2 inhibitor, significantly inhibited the growth and vascularization of murine melanoma xenografts in zebrafish. There was almost no effect on normal vessel formation [143]. Angiogenesis and anti-angiogenic miRNAs have been studied in a zebrafish prostate cancer cell xenograft [144]. Recently, stellettin B, a naturally occurring marine triterpenoid, was tested in a zebrafish xenograft model of glioblastoma. Stellettin B was shown to significantly inhibit angiogenesis in vitro as well as in vivo in zebrafish [145]. There is a recent paper focusing on human melanoma xenotransplantation and the role of interleukin 8 (*CXCL8*) together with *bcl-xL* on cancer cell dissemination and angiogenesis in the zebrafish. The authors suggest that the autocrine CXCL8/CXCR2 signaling pathway can escalate melanoma aggressiveness [146]. These studies have shown that zebrafish is a good in vivo model for rapid identification of inhibitors which could have significance in the development of antiangiogenic cancer therapy.

As we have already discussed in Section 2 of this review, the conservation of hematopoietic programs between human and zebrafish is remarkable. Corkery et al. transplanted human leukemic cancer cell lines into *casper.* The cells, circulating in the embryonic vasculature, were able to proliferate in vivo and survived until 7 dpf in the embryos. The authors have tested treatment with known inhibitors of leukemic cell growth, such as imatinib mesylate, in vivo. There was a significant decrease in the number of leukemic cells in treated groups compared to controls [147]. Another study looked into pathogenesis and inhibition of human leukemic cell growth but added CD34^+^ leukemic blast cells sorted from blood of AML patients. The xenografted cancer cells were able to survive in zebrafish and were inhibited by imatinib and other antileukemic drugs [148]. Patient-derived T-ALL was successfully engrafted in zebrafish where specific drug response was determined in vivo. The authors identified a gain-of-function *NOTCH1* mutation in patient derived T-ALL primary cells. These cells were sensitive to γ-secretase inhibition [149]. Multiple myeloma (MM) has been studied in zebrafish where the authors evaluated various therapeutic agents after transplanting human MM cell lines as well as primary CD138^+^ MM cells derived from patients. The cells were able to survive, grow, and disseminate in *casper* and they responded to inhibitors. Furthermore, patient-derived cells responded well to the same drugs such as the ones used in patients. This way it might be possible to use zebrafish PDX to assess drug efficacy and sensitivity [150]. Cancer progress is often characterized by cell dissemination and subsequent homing to the bone marrow. Sacco et al. xenotransplanted human bone marrow-derived MM cells or MM cell lines. The authors then followed cell homing into the area of caudal hematopoietic tissue (CHT), which is the region of zebrafish embryonal hematopoiesis and could represent a bone marrow-like niche. The cells homing to CHT had differentially expressed genes, regulating for example cell adhesion or angiogenesis [151].

Zebrafish has proven to be a good model for the study of human breast and prostate cancer tumorigenesis and invasion. The lack of genetic models of de novo cancers of this type in zebrafish, because of missing mammary glands and prostate tissue, are compensated by xenotransplantation studies. Many of the following studies use seemingly unrelated types of cancer cell lines, however, the authors are usually trying to find correlations between in vitro and in vivo invasion abilities and general pathogenesis as well as the potential of cancer cells to metastasize in vivo. In the last decade, the most commonly studied types of solid tumors in zebrafish are melanoma [152], breast, prostate, colon, and pancreatic cancers [153] and glioblastoma [154]. Here, we will walk through the course of time and illustrate on the diversity of xenograft studies how zebrafish contributed to our understanding of tumorigenesis and helped to describe new potential therapeutics.

Eguiara et al. have developed a rapid assay for cancer stem-like cell identification in a zebrafish breast cancer xenograft model. Cells, which were first grown in culture in mammospheres, were more invasive in zebrafish embryos than cells grown in a monolayer. Curcumin treated cells showed significantly decreased migration and tumor formation in vivo [25]. It has been shown that the zebrafish genome contains estrogen-responsive genes and that estrogen-related signaling pathways are relevant compared to humans. Therefore, zebrafish can be a model for estrogen-dependent cancer research, and estrogen responsiveness is highly conserved between zebrafish and humans [155]. Ghotra et al. developed a whole animal imaging assay for following cancer metastasis and dissemination in vivo in zebrafish. The behavior of xenografted cancer cells corresponded to findings from rodent models. The authors compared highly and low malignant cell lines of breast, colorectal, and prostate cancer. Their results suggest that E-cadherin silencing by shRNA boosted breast carcinoma cell dissemination. [156]. Breast cancer invasiveness is known to be controlled by the transforming growth factor beta (TGF-β) signaling pathway. Metastatic properties of different breast cancer cell lines were assessed for their invasiveness and malignity after xenotransplantation into zebrafish. Inhibition of TGF-β signaling with TGF-β receptor kinase inhibitors prevented the invasion of cancer cells, which correlated with findings from a mouse metastasis model [157]. TGF-β induced EMT was further investigated in a zebrafish model of breast cancer metastasis. The transcription factors Snail and Slug have been found to be important in the process of EMT regulation. The authors claim that overexpression of Snail and Slug could promote metastasis and the invasion of single cancer cells in vivo [158]. However, this signaling pathway and its effects on cancer cell migration seem to be more complicated. Integrins represent a class of receptor proteins promoting adhesion and cell proliferation. Integrins are interesting therapeutic targets in breast cancer treatment. Disruption of β_1_ integrin mediates cell adhesion, triggers TGF-β signaling, and EMT. It was revealed that the loss of the β_1_ integrin subunit can block breast tumor growth but also enhance the dissemination of tumor cells [159]. A specific prometastatic switch has been described in E-cadherin positive triple-negative breast cancer (TNBC) cells. The balance between miR-200 microRNAs and the transcription factor zinc finger E-box-binding homeobox 2 (ZEB2) appears to be important for TGF-β signaling and modulates cell survival, proliferation, and migration. The authors suggested reconsidering the use of drugs targeting β_1_ integrins in TNBC [159]. The role of bone morphogene proteins (BMP) in breast cancer pathogenesis is less well described than that of TGF-β. BMP signaling is regulated by different Smad proteins located downstream in the signaling pathway. BMP signaling was shown to exert anti-metastatic signals in breast cancer cells. [160].

The CXCR4-CXCL12 signaling axis has also been studied in a zebrafish TNBC xenograft model. As TNBC is a highly aggressive type of breast cancer with limited treatment option it is essential to explore new treatment alternatives. The authors have shown that human cancer cells expressing CXCR4 could recognize zebrafish ligands and as a result, they initiate early metastasis. Chemical inhibition by IT1t, a CXCR4 antagonist, blocked TNBC metastasis and thus CXCR4 was proposed as a new pharmacological target in TNBC [161]. Recently, a further role of CXCR4 signaling was described in tumor–immune cell communication. Specifically, the role of neutrophil motility in the onset of micrometastasis formation was shown to be dependent on CXCR4 signaling [162].

It is widely accepted that the ability of cancer cells for self-renewal, also termed stemness, is a marker of highly proliferative, aggressive, dedifferentiated tumor cells. These cells often overexpress marker genes typically found active in embryonic stem cells, such as SOX2 and OCT4 [163,164]. The role of AKT and SOX2 in breast carcinoma was evaluated in zebrafish. AKT can stabilize SOX2 in breast carcinoma cells and CSCs seem to be dependent on AKT signaling. Therefore, inhibiting AKT might provide a new way of targeting SOX2 positive breast carcinoma cells [165].

Mercatali et al. used PDX from bone metastasis of a breast cancer patient and compared the behavior of PDX to established breast cancer cell lines. Primary cells from patients extravasated from vessels and invaded into the CHT of zebrafish. Therefore, zebrafish might be a good preclinical model to identify breast cancer prognostic markers as well as to predict response to therapy [166].

The most commonly found cancer type in males is prostate cancer. To achieve the best results in prostate cancer treatment, it is desirable to detect it in early stages, when it is prostate-confined. The effect of a nonreceptor spleen tyrosine kinase *SYK* on the dissemination of prostate cancer cells has been studied in a zebrafish and mouse xenograft model. The role of *SYK* in epithelial cancer is divergent. Silencing of *SYK* prevented cancer cell dissemination in vitro and in vivo and pharmacological inhibition of *SYK* led to a similar decrease in cancer invasiveness [167]. In another study, the androgen-dependent LNCaP prostate cancer cell line was xenotransplanted into zebrafish. Administration of exogenous testosterone to LNCaP xenografted zebrafish increased cancer cell proliferation compared to controls. This effect was reversed by the anti-androgen receptor drug, enzalutamide. In contrast, the proliferation of a non-androgen-dependent prostate cancer cell line was not affected by testosterone or enzalutamide treatment. The authors suggested that testosterone administration should be considered in zebrafish xenograft studies of prostate cancer [168]. The invasiveness of the PC3 prostate cancer cell line in zebrafish was recently evaluated and it was suggested as a good model for drug targeted screening for prostate cancer. PC3 cells in this study overexpressed calcitonin receptor (CTR), which led to overall enhanced aggressiveness. The authors have looked for prostate cancer-specific markers to better describe and to detect prostate cancer in patients early [169].

Zebrafish as a model for retinoblastoma [170] and glioblastoma [154,171,172,173,174] has been popular in the last couple of years. Many of these studies highlight the significance of zebrafish in finding novel treatment targets and evaluating cancer inhibitor efficacy in vivo. Glioblastoma is a very heterogeneous and complex type of cancer and is invasive. Despite surgical resection, radiotherapy, and aggressive treatment survival rates are low and the prognosis often negative [175].

Zebrafish has been also recently used as a model of colorectal carcinoma in search of new treatment methods. Marine guanidine alkaloids [176], clinically standard combinatorial therapy [27] as well as bromelain, a pineapple extract [177], have been tested in zebrafish colorectal cancer xenografts. Despite gastric cancers being among the leading cancer types in terms of death rates worldwide, there are not many models of gastric cancer in zebrafish. Recently, two studies described the possibilities to search for a potential treatment of gastric cancer in zebrafish xenografts. Wu et al. have tested chemotherapeutic treatments on primary cancer cells derived from gastric cancer patients in zebrafish. Their PDX model was shown to be reliable and looks promising in searching for personalized treatment [178]. In another study concerning gastric carcinoma Triphala, a traditional medicinal formulation was tested. Triphala has inhibited the growth of xenografted cells and their metastasis, probably through inhibiting the phosphorylation of EGFR/Akt/ERK signaling cascade proteins [179]. Human oral squamous cell carcinoma has also been studied in the zebrafish xenograft model. The authors have investigated the effects of sandensolide, extracted from the herb *Sinularia flexibilis*. Sandensolide can induce apoptosis and could be used as a supporting agent in the treatment of oral cancer [180].

Although zebrafish do not have lungs it can recapitulate cancer-tumor microenvironment interactions reliably. A human non-small-cell lung cancer (NSCLC) xenograft model has been used to study the efficacy as well as the toxicity of three anti-angiogenic drugs. All tested compounds showed anti-angiogenic effects and the inhibition of tumor growth in zebrafish. [181]. Another study revealed the role of autophagy in zebrafish NSCLC xenografts. The authors demonstrated that the combined use of a sub-lethal dose of C2-ceramide and autophagy inhibitors could be promising in NSCLC treatment [182].

The zebrafish model is a good platform for studying rare cancer pathogenesis, for example, Ewing sarcoma (EWS). EWS is rare aggressive childhood cancer. The most commonly found gene fusion in this cancer is *EWSR1–ETS*. New combination therapy was proposed in a zebrafish xenograft model of EWS. Nutlin-3, a *tp53* activator, and YK-4-279, a EWSR1–ETS inhibitor, were shown to be a promising combination therapy for a subset of EWS patients [183].

Transplantation of human cancer cells into zebrafish is an established technique which provides in vivo environment for real-time visualization of cell–cell interactions. Furthermore, zebrafish PDX can support the discovery of potential targeted anti-cancer treatments. Recent successes in zebrafish PDX might help pre-clinical research to significantly shorten the time needed for drug approval, mostly by drug repurposing. Zebrafish models of human cancer xenotransplantation are summarized in Table 4.

### 3.3. Drug Screening in Zebrafish and Its Future as a Pre-clinical Model

Drug screening in zebrafish has become highly popular over the last 10 years. Previously, high-throughput screening for new drugs was basically conducted in vitro in cultured cells and the hits were taken to rodent models where they often failed, proving to be either ineffective or toxic. It is not trivial to assess all biological properties and characteristics of a compound in vitro without having information from the whole animal [19]. Zebrafish embryonal screens can be carried out at a medium (manual cancer cell transplantation) to high throughput (automated yolk sac cancer cell transplantation, de novo cancer, or cancer-related biological pathways) rates, however, the limiting factor is that not all of the steps could be easily automatized. Therefore, compound screens exceeding 1000 compounds have not been done on xenograft zebrafish models, but they were focused more on targeting specific cancer biology related pathways in zebrafish embryos [28,187]. For example, a library of 2000 compounds was tested for inhibition of angiogenesis in zebrafish embryos. Among seven hit compounds, rosuvastatin was further characterized for its antiangiogenic and antineoplastic effects in vitro as well as in vivo in mouse prostate cancer xenografts [188]. In a similar screening study, zebrafish was used to look for neural crest cell growth inhibitors. Leflunomide, an inhibitor of dihydroorotate dehydrogenase, was found as a hit inhibiting also the growth of human melanoma cells, which are originally derived from the embryonic neural crest [189]. Ridges et al. performed a drug screen focused on compounds which are able to eliminate immature T-cells and therefore, prospective for eradicating T-ALL cells as well. For this purpose, they used the *lck:eGFP* transgenic zebrafish line with fluorescently labeled thymic T-cells. After finding primary compound hits which reduced the number of T-cells significantly, these compounds were tested in a human T-ALL cell line. Lenaldekar, a compound with previously unknown biological activity, was identified. Further, its activity was validated in adult T-ALL zebrafish and in a murine xenograft model [190]. A further study looking for potential T-ALL inhibitors in the zebrafish *Myc*-induced T-ALL model was based on hits found in cell culture. An antipsychotic drug targeting protein phosphatase 2A (PP2A), perphenazine, was found to be highly effective in suppressing T-ALL cell growth [191]. Recently, clotrimazol has been discovered as a potential cure for melanoma. The authors further described the effects of clotrimazole co-treatment with other oncogene-specific inhibitors, as Lonafarnib, in vivo in zebrafish [192].

In this way, further potential inhibitors for cancer treatment were identified in zebrafish compound screens, with suggested antineoplastic features mediated by cell cycle delay [193], anti-angiogenic [145,194,195], or anti-lymphangiogenic [196] effects. This approach to compound screening, however, usually requires detailed knowledge about the exact disease pathogenesis and about the target pathway or at least about the biological process which is disordered. Drug treatment is usually done easily by dispensing chemotherapeutics into the fish water as embryos can absorb small molecules dissolved in water. However, it might be difficult to treat zebrafish with water-insoluble drugs, because the carrier solvents for efficient administration may be toxic. For long-term administration of therapeutics in adult zebrafish, a specific protocol for oral gavage has been published [197] and successfully used [186]. Measurement of cancer cell growth can be partially automated as well, by using an automated fluorescent microscopy strategy [156,184]. Using compound libraries containing FDA approved drugs leads to drug repurposing and could accelerate the translation of hits from zebrafish screens to the clinic as in the case of perphenazine [191,198]. Zebrafish has been used as a preclinical model for characterization of nanomedicines as well [199].

The zebrafish cancer xenograft model is an excellent alternative for studying tumor progression and for testing novel therapeutics even in the absence of appropriate transgenic models. Despite lacking tissues such as lung, prostate, or mammary gland, many xenotransplantation studies have proved that zebrafish can recapitulate tumor phenotypes seen in humans, as discussed in the previous section. The effects on tumor microenvironment, as well as the process of metastasis, can be followed real-time and in vivo in zebrafish. The only hurdle which had to be overcome was finding a way for reliable xenotransplantation into adult zebrafish, where the previous immunocompromised models have failed [124,130] and γ-irradiation can be demanding. Cancer stem-like cells (CSCs) have been used for xenotransplantation in adult *casper* immunocompromised by ionizing radiation [184]. In this study, leukemic cells, human prostate cancer cells, as well as liver cancer cells were sorted for high aldehyde dehydrogenase (ALDH) expression. ALDH expression is one of the markers widely used for sorting CSCs from bulk populations of cancer cells. These CSCs were able to rapidly grow in recipients and it was possible to re-transplant them. The authors have established a CSC xenotransplantation model in zebrafish which they suggested as suitable for drug screening purposes [184]. Khan et al. have used busulfan treatment in a recent study and were able to successfully xenograft AML cells and HCC cells into adult zebrafish. The cancer cells survived in the fish for up to 15 days post transplantation [185]. A new double mutant immunodeficient zebrafish model suitable for cancer xenotransplantation was published only recently. This fish has the *prkdc^D3612fs/D3612fs^* mutation together with the mutated *l2rga^Y91fs/Y91fs^* gene [186]. This combination of mutations is currently widely used also in murine xenograft models [200]. Yan et al. have developed specific procedures for adult immunodeficient zebrafish xenotransplantation, maintenance, treatment, and tumor growth evaluation. They have transplanted a wide variety of cancer cell lines as well as patient-derived primary cancer cells and compared their results from xenografted adult fish to results from xenografted mice. Their results seem to be very promising and this model of adult zebrafish xenografts, mainly PDX, might be valuable in the future of cancer research as a reliable pre-clinical model comparable to the mouse [186]. Altogether, the significance of zebrafish as a preclinical model for cancer research is undoubtful. The high reproductive rate of zebrafish and the relatively low-cost maintenance enables high-throughput whole animal screening. There are other papers extensively reviewing zebrafish as a model for cancer cell transplantation [26,33,76] and as a pre-clinical model in drug discovery [28,201].

## 4. Conclusions

Zebrafish has proven to be reliable for modeling and visualizing human cancer cell biology and dynamics, including metastases or tumor tissue neo-angiogenesis, in vivo. Further, the involvement of epigenetic modulators in tumor biology could improve our understanding of such complex diseases as cancer. The availability of transgenic and mutant models, as well as the possibility to transplant cancer cells into zebrafish, provides a wide array of options for studying human cancer. Although zebrafish is a non-mammalian model organism, it has striking evolutionary conservation of disease-related genes and pathways with humans. Searching for novel drugs could be done in vitro at large scale but the effects on the whole living organism might be markedly different. Screening for targeted treatment in zebrafish xenografts could provide new opportunities for anticancer personalized therapy in the future as recent research has shown that zebrafish studies are reliable in modeling human cancer.

## Figures and Tables

**Table 1:** Genetic models of cancer in the zebrafish.

Cancer	Genotype	Zebrafish Background	Reference
Peripheral nerve sheath tumor (PNST)	*tp53^M214K^*	WT	[39]
	*brca2^Q658X^ tp53^M214K^*	WT or *tp53^M214K^*	[47]
PNST, angiosarcoma, leukemia, germ cell tumor	*tp53^del/del^*	CG1 syngeneic zebrafish strain	[40]
Rhabdomyosarcoma (RMS)	*rag2:KRAS^G12D^* *rag2:dsRed2*	WT; *α-actin:GFP*; *tp53^M214K^*	[43,44]
Melanoma	*BRAF^V600E^ tp53^M214K^*	*tp53^M214K^*	[45]
	*BRAF^V600E^ tp53^M214K^*	*crestin:EGFP; tp53^M214K^*	[50]
	*BRAF^V600E^mitfa^vc7^*	*mitfa^vc7^*	[54]
	*hsp70I:GFP-HRAS^G12V^*	N.A.	[51,55]
	*kita:GalTA4,UAS:mCherry* *UAS:eGFP-HRAS^GV12^*	N.A.	[52,55]
	*kita:Gal4TA, UAS:mCherry* *UAS:eGFP-HRAS^GV12^* *UAS:eGFP-jmjd6*	WT or *tp53^M214K^*	[55]
Thyroid cancer	*tg:BRAF^V600E^-pA;tg:TdTomato-pA*	WT	[53]
Pancreatic cancer	*ptf1a:eGFP-KRAS^G12V^*	WT	[56]
	*ptf1a:CRE^ERT2^* *ubb:lox-Nuc-eCFP-stop-lox-GAL4-VP16* *UAS:eGFP-KRAS^G12V^*	N.A.	[57]
Hepatocellular cancer (HCC)	*fabp10a: RPIA; myl7:GFP*	N.A.	[58]
	*fabp10:rtTA2s-M2;TRE2:eGFP-kras^G12V^*	WT or *lepr^+/-^*	[60]
	*fabp10:TA; TRE:Myc; krt4:GFP* *fabp10:TA; TRE:xmrk; krt4:GFP*	WT	[61]
Intestinal tumors	*pDs-ifabp:LexPR-Lexop:eGFP-kras^V12^*	N.A.	[59]
	*5×UAS:EGFP-P2A-kras^G12D^* *fabp10a:mCherry* *fabp10a:mCherry-P2A-cyp7a1* + various Gal4 lines	WT or *cyp7a1^−5^*	[62]
Testicular tumor	*brca2^Q658X^*	WT	[48]
T-cell acute lymphoid leukemia (T-ALL)	*rag2:mMyc* *rag2:GFP* *rag2:dsRed2*	WT	[42,43]
	*rag2:loxP-dsRED2-loxP-eGFP-mMyc*	WT	[66]
	*spi1:tel-jak2a*	WT	[72]
Acute lymphoid leukemia (AML)	*hsp70:AML1-ETO*	WT	[68,69]
	*spi1:MYST3/NCOA2-eGFP*	N.A.	[70]
	*pHsFLT3-WT-T2a-eGFP* *pHsFLT3-ITD-T2a-eGFP* *FLT3-ITD-T2a-mRFP*	WT	[74]
Chronic myeloid leukemia (CML)	*spi1:tel-jak2a*	WT	[71,72]
Myelodysplastic syndrome (MDS)	*tet2^-/-^*	*cmyb:eGFP*; *cd41:eGFP*	[75]

WT: Wild type; N.A: Not Available.

**Table 2:** Epigenetic regulators in zebrafish cancer.

Cancer	Zebrafish Genotype	Epigenetic Regulator	Function	Reference
RMS	*rag2*-*hKRAS^G12D^*	*SUV39H1*	Tumor suppressor	[80]
Melanoma	*BRAF^V600E^ tp53^M214K^*	*SETDB1*	Oncogene	[81]
AML	*NUP98-HOXA9*	*dnmt1*	Oncogene	[82]
Retinoblastoma	*rb1/rb1*	more than 170 tested e.g., *hdac1, rbbp4*	Oncogenes	[36]

**Table 3:** Cancer allograft transplantation models in zebrafish.

Transplanted Cancer Type		Developmental Stage	Injection Site	Reference
**Primary cells**	T-ALL	Adult	Intraperitoneal cavity	[42,66,124,127]
	RMS	Adult	Intraperitoneal cavity	[124,127]
	Melanoma	Adult	Intraperitoneal cavity	[124]
	T-ALL, RMS, melanoma, neuroblastoma	Adult	Intraperitoneal cavity, retro-orbital, intramuscular	[129,130]
	Melanoma	Adult	N.A.	[131]
**ZMELs**	Melanoma	Adult 48 h post-fertilization (hpf)	Subcutaneous Circulation (duct of Cuvier)	[131]
		Adult	Retro-orbital Intravenous (cardinal vein)	[132]
		48 hpf	Circulation	[133]

**Table 4:** Human cancer xenograft transplantation models in zebrafish.

Transplanted Cancer Type		Developmental Stage	Injection Site	Reference
**Cell lines**	Melanoma	Blastula	Blastodisc	[134]
	Melanoma (uveal and cutaneous)	Blastula	N.A.	[135]
	Melanoma and colorectal cancer	48 h post-fertilization (hpf)	Yolk sac; hindbrain ventricle; circulation	[136]
	Uveal melanoma	48 hpf	Yolk sac	[152]
	Melanoma	48 hpf	Yolk sac	[146]
	Colorectal cancer	48 hpf	Yolk sac	[139]
	Colorectal cancer	48 hpf	Yolk sac	[27,176,177]
	Pancreatic cancer	48 hpf	Yolk sac	[140]
	Melanoma, adenocarcinoma, triple negative breast cancer (TNBC) and ovarian cancer	48 hpf	Yolk sac, proximity of subintestinal veins (SIV)	[141,142]
	Colorectal cancer, melanoma (both murine)	48 hpf	Yolk sac	[143]
	Prostate cancer	48 hpf	Yolk sac	[144,167]
	Prostate cancer, androgen dependent and independent	48 hpf	Yolk sac	[168]
	Prostate cancer	48 hpf	Subcutaneous, above yol sack	[169]
	Breast, prostate, colon, pancreatic cancer, fibrosarcoma	48 hpf	Yolk sac	[153]
	Breast cancer	48 hpf	Yolk sac	[25]
	Breast, prostate, colorectal cancer	48 hpf	Yolk sac	[156]
	Breast cancer, non-invasive and metastatic	48 hpf	Duct of Cuvier	[157]
	Breast cancer	48 hpf	Duct of Cuvier	[158]
	Breast cancer	48 hpf	Yolk sac	[159]
	Breast adenocarcinoma and TNBC	48 hpf	Duct of Cuvier	[161]
	TNBC and prostate cancer	48 hpf	Duct of Cuvier	[162]
	Breast cancer	48 hpf	Yolk sac	[165]
	Breast cancer and TNBC	48 hpf	Duct of Cuvier	[166]
	TNBC	48 hpf	Duct of Cuvier	[165]
	AML, CML	48 hpf	Yolk sac	[147]
**Cell lines**	AML, T-ALL	48 hpf	Posterior cardinal vein (PCV)	[148]
	T-ALL	48 hpf	Yolk sac	[149]
	Multiple myeloma (MM)	48 hpf	Yolk sac	[150]
	MM, Waldenstrom’s macroglobulinemia, TNBC	48 hpf	Pericardium	[151]
	CML, HCC, prostate cancer (sorted for cancer stem cells)	48 hpf Adult	Yolk sac Trunk near dorsal aorta	[184]
	AML, HCC	48 hpf Adult	Yolk sac Trunk near dorsal aorta; heart	[185]
	Retinoblastoma	48 hpf	Vitreous cavity	[170]
	Glioblastoma	52 hpf	Yolk sack; brain	[154]
	Glioblastoma	36 hpf	Hindbrain	[171]
	Glioblastoma	72 hpf	Brain	[172]
	Glioblastoma and colon cancer	Blastula	Blastoderm	[174]
	Gastrointestinal tumors – pancreas, stomach, colon	48 hpf	Yolk sac; liver	[140]
	Gastric cancer	48 hpf	Yolk sac	[178,179]
	Oral squamous cell carcinoma	48 hpf	Yolk sac	[180]
	Non-small-cell lung cancer (NSCLC)	48 hpf	Yolk sac	[181]
	NCSLC	48 hpf	N.A.	[182]
	Ewing sarcoma (EWS)	48 hpf Juvenile (35 dpf)	Yolk sac Eye vessels	[183]
	Various types of human cancer	Adult	Intraperitoneal cavity Peri-ocular muscle	[186]
**PDX**	AML blast cells	48 hpf	PCV	[148]
	T-ALL from bone marrow	48 hpf	Yolk sac	[149]
	MM cells from plasma	48 hpf	Yolk sac	[150]
	MM cells from bone marrow	48 hpf	Pericardium	[151]
	Glioblastoma	36 hpf	Brain	[173]
	Glioblastoma	blastula	Blastoderm	[174]
	Gastric cancer	48 hpf	Yolk sac	[178]
	Glioblastoma, melanoma, breast cancer, RMS	Adult	Peri-ocular muscle	[186]
